# Crystal structure of 2-cyclo­hexyl-1,3-thia­zolo[4,5-*b*]pyridine

**DOI:** 10.1107/S2056989015019106

**Published:** 2015-10-17

**Authors:** Gamal A. El-Hiti, Keith Smith, Amany S. Hegazy, Mansour D. Ajarim, Benson M. Kariuki

**Affiliations:** a Cornea Research Chair, Department of Optometry, College of Applied Medical Sciences, King Saud University, PO Box 10219, Riyadh 11433, Saudi Arabia; b School of Chemistry, Cardiff University, Main Building, Park Place, Cardiff CF10 3AT, Wales; c Criminal Evidence, Ministry of Interior, Riyadh 11632, PO Box 86985, Saudi Arabia

**Keywords:** crystal structure, cyclo­hexa­ne, thia­zolo­pyridine derivatives, thia­zolo[4,5-*b*]pyridine

## Abstract

In the title compound, C_12_H_14_N_2_S, the cyclo­hexane ring adopts a chair conformation with the exocyclic C—C bond in an equatorial orientation. The mean plane through the cyclo­hexane ring (all atoms) is twisted from the thia­zolo­pyridine ring system (r.m.s. deviation = 0.013 Å) by 39.57 (6)°. In the crystal, mol­ecules form (100) sheets, although there are no specific directional inter­actions between them. The crystal stucture was refined as a two-component perfect twin.

## Related literature   

For background to the uses of thia­zolo­pyridine derivatives, see: Leysen *et al.* (1984 ▸). For a related structure reported by us and further references, see: El-Hiti *et al.* (2015 ▸). For the first report of this compound and spectroscopic data, see: Smith *et al.* (1995 ▸).
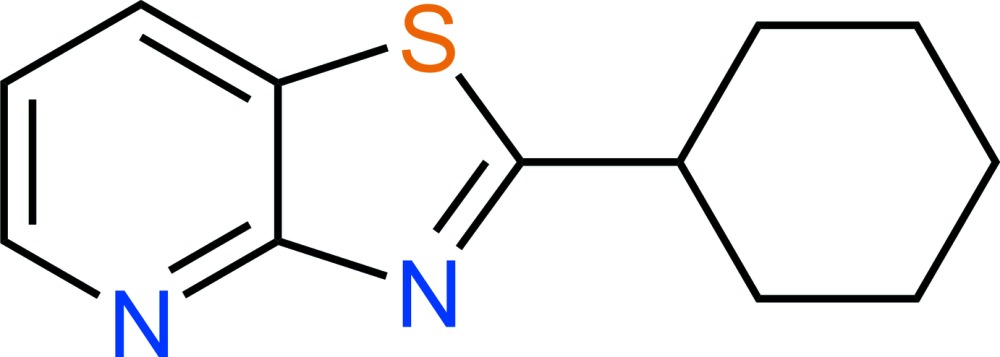


## Experimental   

### Crystal data  

C_12_H_14_N_2_S
*M* *_r_* = 218.31
Monoclinic, 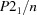

*a* = 7.8884 (5) Å
*b* = 11.8079 (7) Å
*c* = 12.2134 (6) Å
β = 100.589 (6)°
*V* = 1118.25 (11) Å^3^
*Z* = 4
Cu *K*α radiation
μ = 2.29 mm^−1^
*T* = 293 K
0.27 × 0.17 × 0.14 mm

### Data collection  

Agilent SuperNova Dual Source diffractometer with an Atlas detector
Absorption correction: multi-scan (*CrysAlis PRO*; Agilent, 2014 ▸) *T*_min_ = 0.592, *T*_max_ = 1.000
7328 measured reflections
3913 independent reflections
3429 reflections with *I* > 2σ(*I*)
*R*_int_ = 0.015

### Refinement  

*R*[*F* ^2^ > 2σ(*F* ^2^)] = 0.063
*wR*(*F* ^2^) = 0.180
*S* = 1.03
3913 reflections
136 parameters
H-atom parameters constrained
Δρ_max_ = 0.30 e Å^−3^
Δρ_min_ = −0.29 e Å^−3^

### 

Data collection: *CrysAlis PRO* (Agilent, 2014 ▸); cell refinement: *CrysAlis PRO*; data reduction: *CrysAlis PRO*; program(s) used to solve structure: *SHELXS97* (Sheldrick, 2008 ▸); program(s) used to refine structure: *SHELXL2013* (Sheldrick, 2015 ▸); molecular graphics: *ORTEP-3 for Windows* (Farrugia, 2012 ▸); software used to prepare material for publication: *WinGX* (Farrugia, 2012 ▸) and *CHEMDRAW Ultra* (Cambridge Soft, 2001 ▸).

## Supplementary Material

Crystal structure: contains datablock(s) I, New_Global_Publ_Block. DOI: 10.1107/S2056989015019106/hb7518sup1.cif

Structure factors: contains datablock(s) I. DOI: 10.1107/S2056989015019106/hb7518Isup2.hkl

Supporting information file. DOI: 10.1107/S2056989015019106/hb7518Isup3.cml

**Fig. 1.**
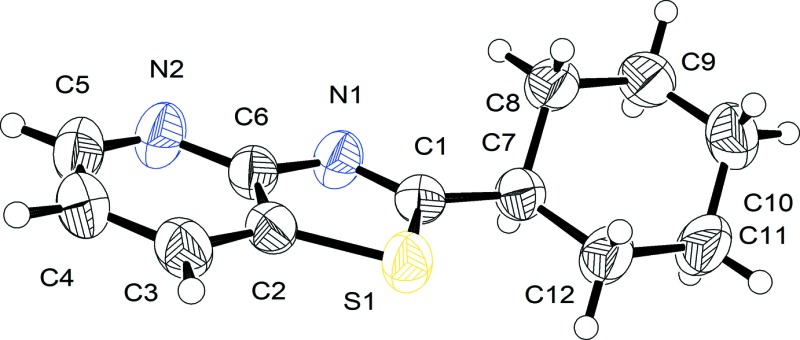
The asymmetric unit of C12H14N2S with 50% probability displacement ellipsoids for nonhydrogen atoms.

**Fig. 2.**
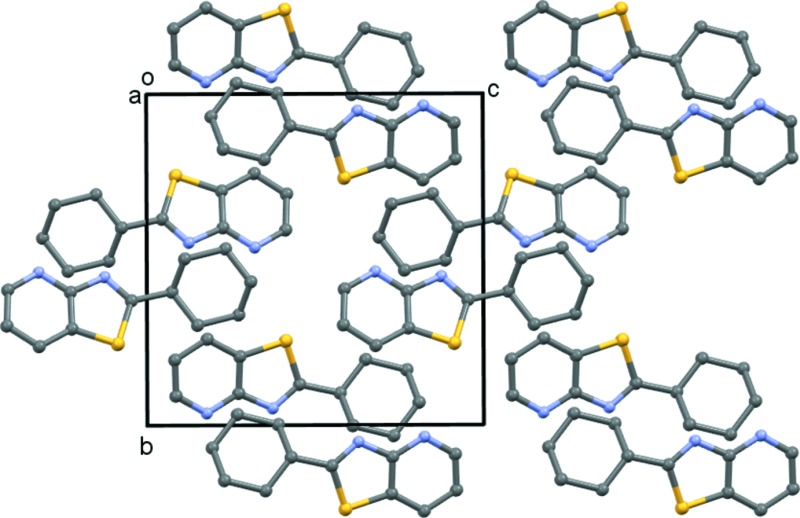
Crystal packing viewed along the a axis.

12 14 2 . DOI: 10.1107/S2056989015019106/hb7518fig1.tif
The asymmetric unit of C_12_H_14_N_2_S with 50% probability displacement ellipsoids for nonhydrogen atoms.

a . DOI: 10.1107/S2056989015019106/hb7518fig2.tif
Crystal packing viewed along the *a* axis.

CCDC reference: 1430576

Additional supporting information:  crystallographic information; 3D view; checkCIF report

## supplementary crystallographic information

### S1. Chemical context

Thia­zolo­pyridine derivatives have been reported to exhibit inter­esting biological activities (Leysen *et al.*, 1984). As part of our ongoing studies in this area (El-Hiti *et al.*, 2015), we now report the structure of the title compound.

### S2. Structural commentary

The asymmetric unit comprises one molecule of C_12_H_14_N_2_S (Fig. 1). The cyclo­hexane ring is in the chair conformation in the molecule. The least squares plane through the cyclo­hexane ring is twisted from the thia­zolo­pyridine group by 39.57 (6)°. In the crystal structure, the molecular axes are aligned along [001] (Fig. 2).

### S3. Synthesis and crystallization

2-Cyclo­hexyl-1,3-thia­zolo[4,5-*b*]pyridine was obtained in qu­anti­tative yield from acid hydrolysis (HCl, 5 M) of 3-(diiso­propyl­amino­thio­carbonyl­thio)-2-(cyclo­hexyl­carbonyl­amino)­pyridine under reflux for 5 h (Smith *et al.*, 1995). The compound may also be synthesized in 94% yield from acid hydrolysis (5 M HCl, 5 h reflux) of 3-(diiso­propyl­amino­thio­carbonyl­thio)-2-(*bis*(cyclo­hexyl­carbony)lamino) pyridine (Smith *et al.*, 1995). Crystallization of the crude product from di­ethyl ether gave the title compound as colourless crystals. Spectroscopic and analytical data are consistent with those reported (Smith *et al.*, 1995).

### S4. Refinement details

The data were twinned and HKLF5 in Shelxl 2013 was used. H atoms were positioned geometrically and refined using a riding model with *U*_iso_(H) constrained to be 1.2 times *U*_eq_ for the atom it is bonded to.

### Crystal data

**Table:**

C_12_H_14_N_2_S	*F*(000) = 464
*M**_r_* = 218.31	*D*_x_ = 1.297 Mg m^−^^3^
Monoclinic, *P*2_1_/*n*	Cu *K*α radiation, λ = 1.54184 Å
*a* = 7.8884 (5) Å	Cell parameters from 1721 reflections
*b* = 11.8079 (7) Å	θ = 6.2–73.9°
*c* = 12.2134 (6) Å	µ = 2.29 mm^−^^1^
β = 100.589 (6)°	*T* = 293 K
*V* = 1118.25 (11) Å^3^	Needle, colourless
*Z* = 4	0.27 × 0.17 × 0.14 mm

### Data collection

**Table:**

Agilent SuperNova Dual Source diffractometer with an Atlas detector	3429 reflections with *I* > 2σ(*I*)
ω scans	*R*_int_ = 0.015
Absorption correction: multi-scan (*CrysAlis PRO*; Agilent, 2014)	θ_max_ = 74.2°, θ_min_ = 5.3°
*T*_min_ = 0.592, *T*_max_ = 1.000	*h* = −9→9
7328 measured reflections	*k* = −14→14
3913 independent reflections	*l* = −15→15

### Refinement

**Table:**

Refinement on *F*^2^	0 restraints
Least-squares matrix: full	Hydrogen site location: inferred from neighbouring sites
*R*[*F*^2^ > 2σ(*F*^2^)] = 0.063	H-atom parameters constrained
*wR*(*F*^2^) = 0.180	*w* = 1/[σ^2^(*F*_o_^2^) + (0.1345*P*)^2^ + 0.113*P*] where *P* = (*F*_o_^2^ + 2*F*_c_^2^)/3
*S* = 1.03	(Δ/σ)_max_ = 0.001
3913 reflections	Δρ_max_ = 0.30 e Å^−^^3^
136 parameters	Δρ_min_ = −0.29 e Å^−^^3^

### Special details

**Table:**

Experimental. Absorption correction: CrysAlisPro, Agilent Technologies, Version 1.171.37.33 (release 27-03-2014 CrysAlis171 .NET) (compiled Mar 27 2014,17:12:48) Empirical absorption correction using spherical harmonics, implemented in SCALE3 ABSPACK scaling algorithm. Empirical absorption correction using spherical harmonics, implemented in SCALE3 ABSPACK scaling algorithm.
Geometry. All e.s.d.'s (except the e.s.d. in the dihedral angle between two l.s. planes) are estimated using the full covariance matrix. The cell e.s.d.'s are taken into account individually in the estimation of e.s.d.'s in distances, angles and torsion angles; correlations between e.s.d.'s in cell parameters are only used when they are defined by crystal symmetry. An approximate (isotropic) treatment of cell e.s.d.'s is used for estimating e.s.d.'s involving l.s. planes.
Refinement. Refined as a 2-component perfect twin.

### Fractional atomic coordinates and isotropic or equivalent isotropic displacement parameters (Å^2^)

**Table:**

	*x*	*y*	*z*	*U*_iso_*/*U*_eq_	
C1	0.2814 (3)	0.12366 (19)	0.54649 (18)	0.0467 (5)	
C2	0.2459 (3)	0.20807 (19)	0.72356 (18)	0.0467 (5)	
C3	0.2223 (4)	0.2609 (2)	0.8209 (2)	0.0561 (6)	
H3	0.1818	0.3349	0.8212	0.067*	
C4	0.2619 (4)	0.1982 (3)	0.9169 (2)	0.0634 (7)	
H4	0.2461	0.2291	0.9844	0.076*	
C5	0.3249 (4)	0.0897 (3)	0.9136 (2)	0.0659 (7)	
H5	0.3505	0.0501	0.9805	0.079*	
C6	0.3115 (3)	0.0972 (2)	0.72741 (18)	0.0472 (5)	
C7	0.2823 (3)	0.0976 (2)	0.42621 (18)	0.0501 (5)	
H7	0.3994	0.0741	0.4202	0.060*	
C8	0.1612 (4)	−0.0018 (2)	0.3883 (2)	0.0616 (7)	
H8A	0.1972	−0.0671	0.4350	0.074*	
H8B	0.0450	0.0181	0.3967	0.074*	
C9	0.1620 (4)	−0.0321 (2)	0.2671 (2)	0.0654 (7)	
H9A	0.2758	−0.0588	0.2600	0.079*	
H9B	0.0805	−0.0929	0.2443	0.079*	
C10	0.1149 (4)	0.0680 (3)	0.1922 (2)	0.0706 (8)	
H10A	−0.0030	0.0901	0.1937	0.085*	
H10B	0.1218	0.0471	0.1163	0.085*	
C11	0.2341 (5)	0.1671 (3)	0.2281 (2)	0.0856 (11)	
H11A	0.1964	0.2320	0.1811	0.103*	
H11B	0.3501	0.1478	0.2188	0.103*	
C12	0.2352 (5)	0.1982 (2)	0.3501 (2)	0.0709 (8)	
H12A	0.3176	0.2587	0.3723	0.085*	
H12B	0.1220	0.2257	0.3577	0.085*	
N1	0.3309 (3)	0.05196 (17)	0.62604 (16)	0.0529 (5)	
N2	0.3519 (3)	0.0371 (2)	0.82184 (17)	0.0616 (6)	
S1	0.20709 (8)	0.25440 (5)	0.58736 (5)	0.0545 (3)	

### Atomic displacement parameters (Å^2^)

**Table:**

	*U*^11^	*U*^22^	*U*^33^	*U*^12^	*U*^13^	*U*^23^
C1	0.0509 (11)	0.0426 (11)	0.0457 (11)	−0.0033 (9)	0.0065 (9)	−0.0001 (8)
C2	0.0516 (11)	0.0423 (11)	0.0436 (11)	−0.0036 (9)	0.0021 (8)	−0.0008 (9)
C3	0.0664 (15)	0.0505 (13)	0.0496 (13)	−0.0023 (11)	0.0058 (11)	−0.0090 (10)
C4	0.0767 (17)	0.0704 (18)	0.0419 (12)	−0.0073 (13)	0.0076 (11)	−0.0079 (11)
C5	0.0794 (17)	0.0733 (18)	0.0425 (13)	0.0014 (13)	0.0047 (11)	0.0092 (11)
C6	0.0507 (12)	0.0475 (12)	0.0425 (11)	0.0013 (9)	0.0060 (8)	0.0012 (9)
C7	0.0547 (13)	0.0510 (13)	0.0453 (11)	−0.0017 (9)	0.0107 (9)	−0.0023 (9)
C8	0.0855 (18)	0.0504 (13)	0.0502 (13)	−0.0138 (13)	0.0160 (12)	−0.0044 (10)
C9	0.0846 (18)	0.0552 (15)	0.0563 (15)	−0.0118 (13)	0.0121 (12)	−0.0137 (11)
C10	0.0842 (19)	0.0764 (19)	0.0473 (13)	0.0011 (15)	0.0020 (12)	−0.0089 (13)
C11	0.145 (3)	0.0712 (19)	0.0416 (13)	−0.030 (2)	0.0189 (15)	0.0008 (12)
C12	0.116 (2)	0.0519 (15)	0.0442 (13)	−0.0148 (15)	0.0133 (13)	−0.0002 (11)
N1	0.0672 (12)	0.0472 (10)	0.0450 (10)	0.0074 (9)	0.0121 (8)	0.0036 (8)
N2	0.0795 (14)	0.0581 (13)	0.0467 (11)	0.0120 (11)	0.0104 (9)	0.0103 (9)
S1	0.0753 (5)	0.0424 (4)	0.0425 (4)	0.0053 (2)	0.0017 (3)	0.0013 (2)

### Geometric parameters (Å, º)

**Table:**

C1—N1	1.294 (3)	C7—H7	0.9800
C1—C7	1.502 (3)	C8—C9	1.524 (4)
C1—S1	1.756 (2)	C8—H8A	0.9700
C2—C3	1.385 (3)	C8—H8B	0.9700
C2—C6	1.405 (3)	C9—C10	1.499 (4)
C2—S1	1.724 (2)	C9—H9A	0.9700
C3—C4	1.374 (4)	C9—H9B	0.9700
C3—H3	0.9300	C10—C11	1.515 (4)
C4—C5	1.378 (4)	C10—H10A	0.9700
C4—H4	0.9300	C10—H10B	0.9700
C5—N2	1.332 (3)	C11—C12	1.534 (4)
C5—H5	0.9300	C11—H11A	0.9700
C6—N2	1.342 (3)	C11—H11B	0.9700
C6—N1	1.383 (3)	C12—H12A	0.9700
C7—C12	1.512 (4)	C12—H12B	0.9700
C7—C8	1.530 (3)		
			
N1—C1—C7	123.0 (2)	H8A—C8—H8B	108.0
N1—C1—S1	115.61 (17)	C10—C9—C8	111.3 (2)
C7—C1—S1	121.33 (17)	C10—C9—H9A	109.4
C3—C2—C6	119.8 (2)	C8—C9—H9A	109.4
C3—C2—S1	131.07 (19)	C10—C9—H9B	109.4
C6—C2—S1	109.08 (17)	C8—C9—H9B	109.4
C4—C3—C2	116.4 (2)	H9A—C9—H9B	108.0
C4—C3—H3	121.8	C9—C10—C11	111.2 (2)
C2—C3—H3	121.8	C9—C10—H10A	109.4
C3—C4—C5	120.2 (2)	C11—C10—H10A	109.4
C3—C4—H4	119.9	C9—C10—H10B	109.4
C5—C4—H4	119.9	C11—C10—H10B	109.4
N2—C5—C4	124.9 (2)	H10A—C10—H10B	108.0
N2—C5—H5	117.6	C10—C11—C12	111.0 (3)
C4—C5—H5	117.6	C10—C11—H11A	109.4
N2—C6—N1	121.2 (2)	C12—C11—H11A	109.4
N2—C6—C2	123.3 (2)	C10—C11—H11B	109.4
N1—C6—C2	115.5 (2)	C12—C11—H11B	109.4
C1—C7—C12	113.3 (2)	H11A—C11—H11B	108.0
C1—C7—C8	109.77 (19)	C7—C12—C11	111.5 (3)
C12—C7—C8	110.3 (2)	C7—C12—H12A	109.3
C1—C7—H7	107.8	C11—C12—H12A	109.3
C12—C7—H7	107.8	C7—C12—H12B	109.3
C8—C7—H7	107.8	C11—C12—H12B	109.3
C9—C8—C7	111.1 (2)	H12A—C12—H12B	108.0
C9—C8—H8A	109.4	C1—N1—C6	110.6 (2)
C7—C8—H8A	109.4	C5—N2—C6	115.3 (2)
C9—C8—H8B	109.4	C2—S1—C1	89.19 (10)
C7—C8—H8B	109.4		
