# Supplementary figures and images for: Crystal structure of 2-cyclo­hexyl-1,3-thia­zolo[4,5-b]pyridine
Source: Acta Crystallogr E Crystallogr Commun. 2015 Oct 17;71(Pt 11):o866. doi: 10.1107/S2056989015019106 (PMC4645017; doi:10.1107/S2056989015019106)

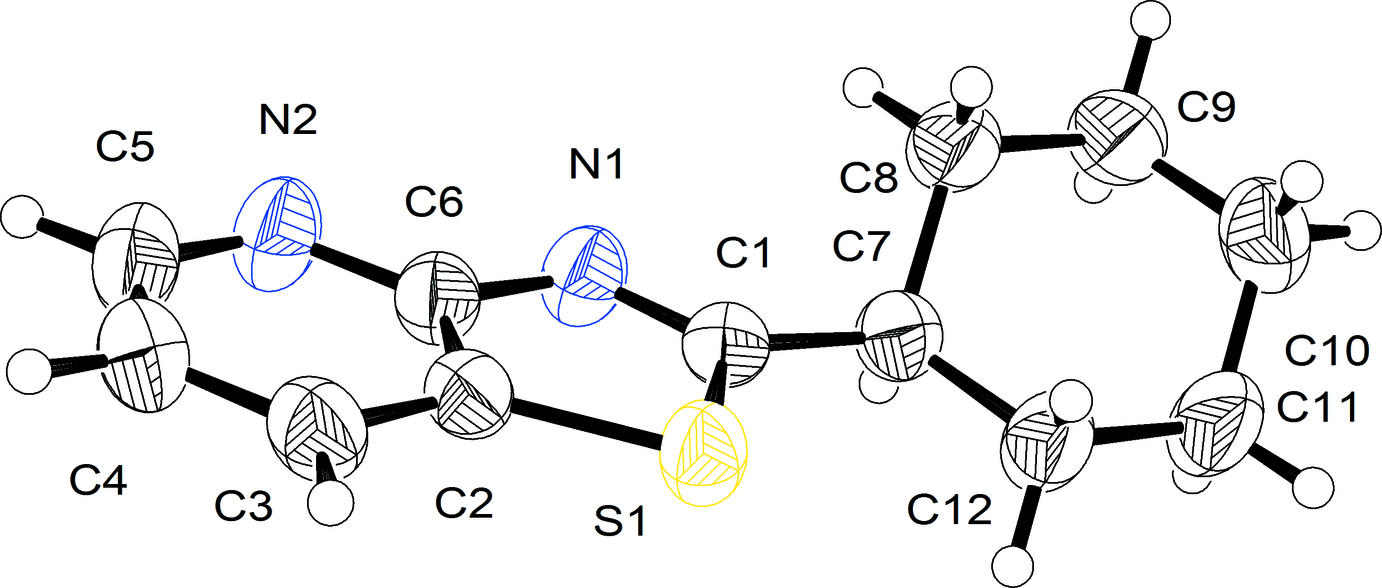

Supplement: Supplementary file 4 [file e-71-0o866-fig1.tif]

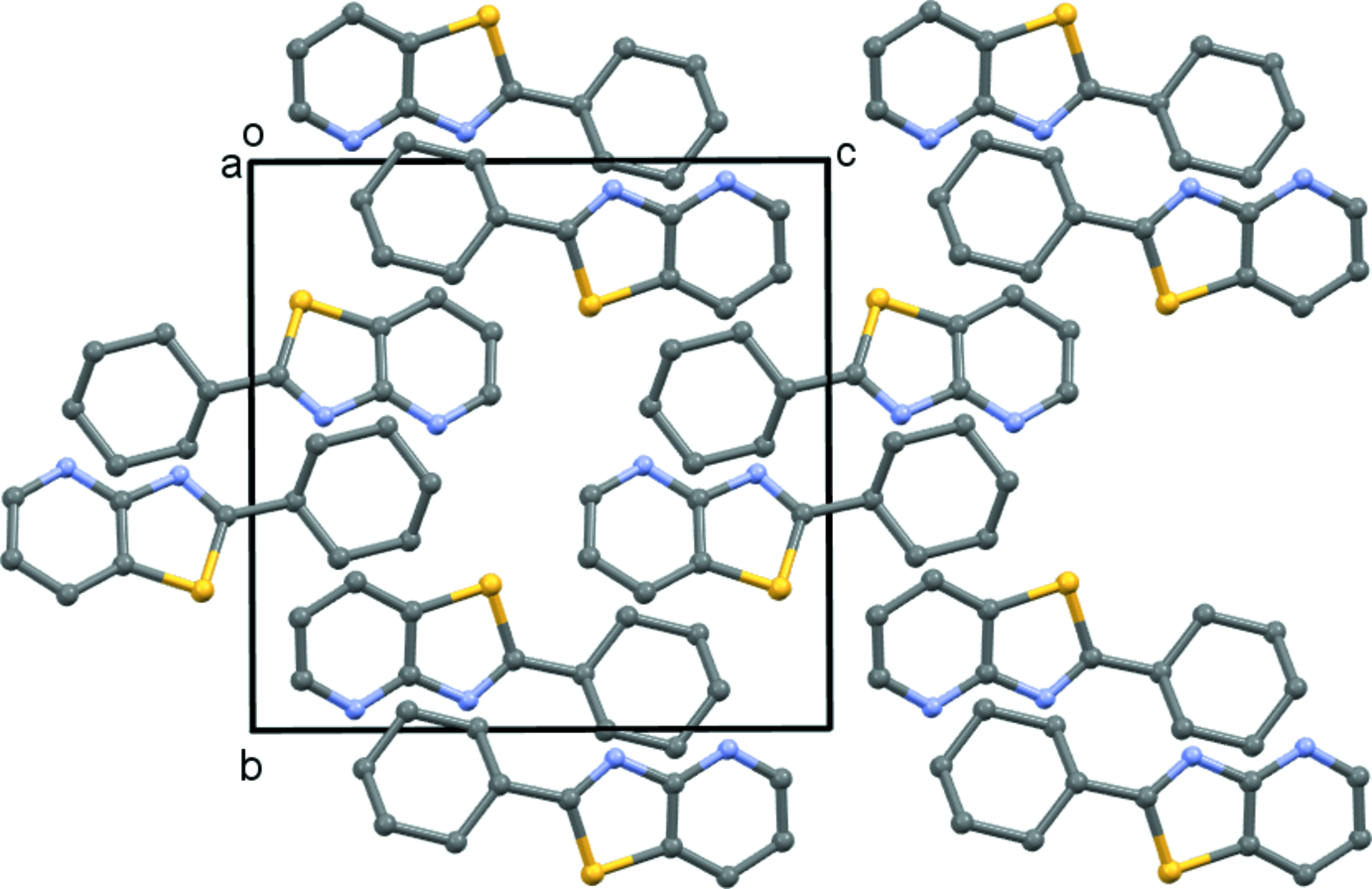

Supplement: Supplementary file 5 [file e-71-0o866-fig2.tif]
